# Deep breathing couples CSF and venous flow dynamics

**DOI:** 10.1038/s41598-022-06361-x

**Published:** 2022-02-16

**Authors:** Jost M. Kollmeier, Lukas Gürbüz-Reiss, Prativa Sahoo, Simon Badura, Ben Ellebracht, Mathilda Keck, Jutta Gärtner, Hans-Christoph Ludwig, Jens Frahm, Steffi Dreha-Kulaczewski

**Affiliations:** 1 Biomedizinische NMR, Max-Planck-Institut für multidisziplinäre Naturwissenschaften, 37077 Göttingen, Germany; 2 Division of Pediatric Neurology, Department of Pediatrics and Adolescent Medicine, University Medical Center Göttingen, 37075 Göttingen, Germany; 3 Division of Pediatric Neurosurgery, Department of Neurosurgery, University Medical Center Göttingen, 37075 Göttingen, Germany; 4 DZHK (German Center for Cardiovascular Research), Partner Site, Göttingen, Germany

**Keywords:** Diseases of the nervous system, Neurological disorders, Hydrocephalus, Magnetic resonance imaging

## Abstract

Venous system pathologies have increasingly been linked to clinically relevant disorders of CSF circulation whereas the exact coupling mechanisms still remain unknown. In this work, flow dynamics of both systems were studied using real-time phase-contrast flow MRI in 16 healthy subjects during normal and forced breathing. Flow evaluations in the aqueduct, at cervical level C3 and lumbar level L3 for both the CSF and venous fluid systems reveal temporal modulations by forced respiration. During normal breathing cardiac-related flow modulations prevailed, while forced breathing shifted the dominant frequency of both CSF and venous flow spectra towards the respiratory component and prompted a correlation between CSF and venous flow in the large vessels. The average of flow magnitude of CSF was increased during forced breathing at all spinal and intracranial positions. Venous flow in the large vessels of the upper body decreased and in the lower body increased during forced breathing. Deep respiration couples interdependent venous and brain fluid flow—most likely mediated by intrathoracic and intraabdominal pressure changes. Further insights into the driving forces of CSF and venous circulation and their correlation will facilitate our understanding how the venous system links to intracranial pressure regulation and of related forms of hydrocephalus.

## Introduction

Recent studies of cerebrospinal fluid (CSF) circulation, pathways and dynamics have rigorously challenged century-old classical views^1–5^. Still, our understanding about the disturbances of CSF circulation underlying the various forms of hydrocephalus and syringomyelia across all ages is vastly incomplete^6^. Hence, detailed insights into regulatory forces of CSF dynamics are of imminent clinical importance to better comprehend the pathogeneses and, more importantly, to derive the most specific therapeutic strategies.

The advent of real-time phase-contrast flow magnetic resonance imaging (MRI), a technique independent of any physiological gating^7^, revealed the influence of respiration on the CSF movement in addition to its well-known cardiac-driven flow^8^. In particular, forced inspiration has been identified as the dominant driver that prompts an upward surge of CSF from the lumbar region along the entire spinal canal, into the cranial vault and towards the subarachnoid and brain ventricular spaces^9^. During exhalation CSF was observed to move downward to varying extent. Cardiac-driven CSF pulsations, most prevalent in cervical regions in close proximity to the heart, are superimposed by a dominating, high-amplitude flow component triggered by forced respiration^9–11^. Furthermore, the inspiratory lowering of the intrathoracic pressure governs venous drainage out of the head/neck region as a prerequisite to ensure adequate preload of the heart. Real-time flow MRI demonstrated fluctuations of outflow in the cervical epidural veins dependent on the strength of inhalation^11^. These latest insights point to a tightly regulated equilibrium between CSF and venous systems, that might play an essential role in regulating the intracranial volume in accordance with the Monro–Kellie doctrine^12^.

The notion of a dynamic interplay between CSF and venous flow is in line with an increasingly popular holistic view on brain fluids^13,14^. Mounting clinical evidence substantiates the importance of the cerebral venous systems for maintaining intracranial pressure (ICP) and hence its role in a broad spectrum of neurological diseases like communicating hydrocephalus and myelopathy^15,16^. A high prevalence of extra- and intracranial venous flow obstruction was found in patients with idiopathic intracranial hypertension (IIH)^17^. However, the physiological mechanisms, which link CSF and veins, are still poorly understood.

Our study aimed (i) to provide further experimental support that respiration couples CSF and venous fluid systems, (ii) to include both normal free breathing and deep respiration in the study protocol, and (iii) to extend such measurements to the lower body.

## Results

### Flow dynamics during normal and forced breathing

**Figure 1:**
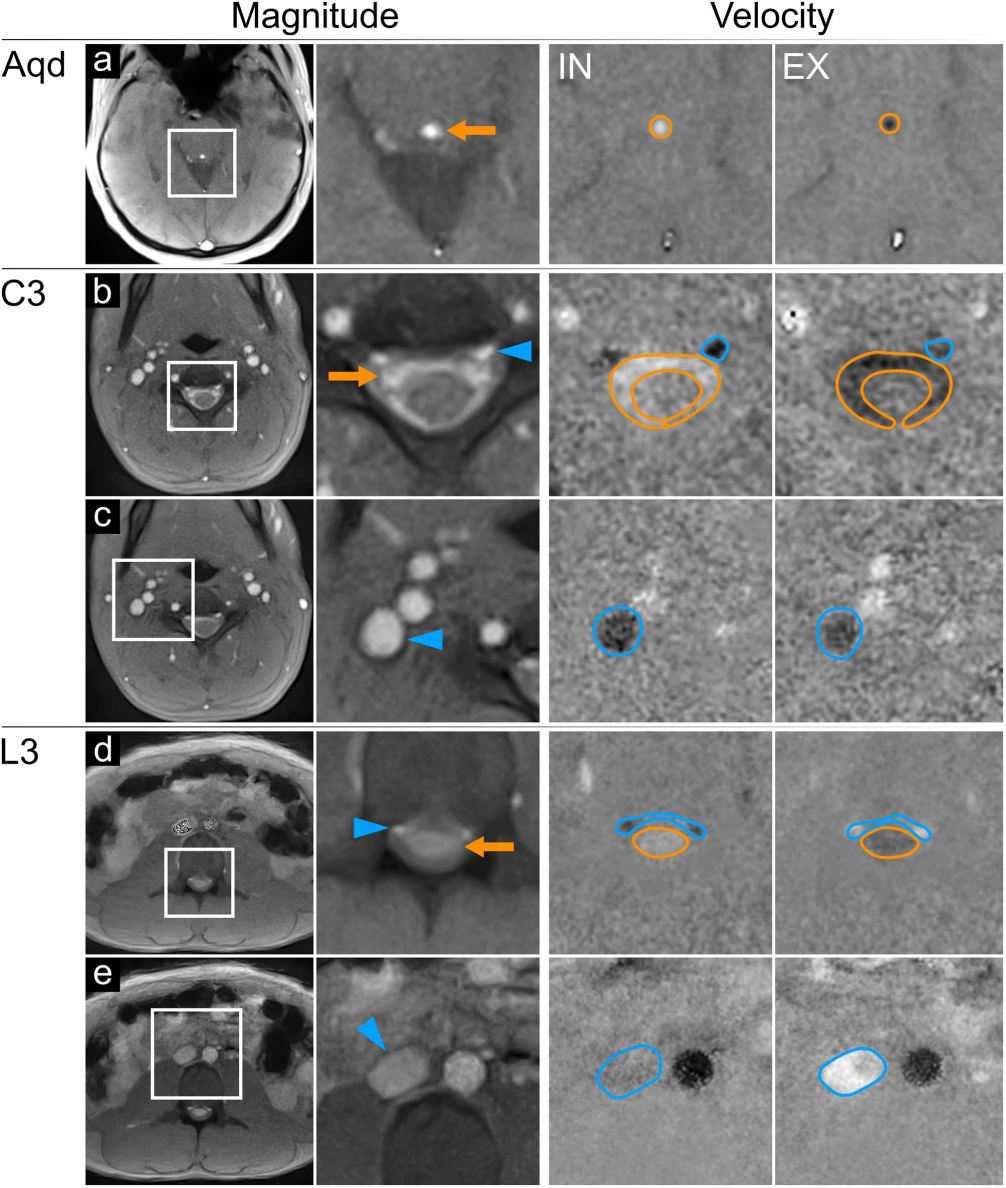
Regions of interest for CSF and venous flow analyses. Magnitude images (left columns) in normal and magnified views at the aqueduct. **Aqd** ((**a**); subject #2), spinal cervical level **C3** ((**b,c**); subject #10), and lumbar level **L3** ((**d,e**); subject #5). CSF flow (orange arrows) appears as bright signal in the Aqd (**a**), the subarachnoid spaces at C3 (**b**) and L3 (**d**). Flow in cervical (**b**) and lumbar (**d**) EV (blue arrow heads) and in IJV (**c**) and IVC (**e**). **Velocity maps** (right columns) during forced inspiration **IN** and expiration **EX**. Magnified maps indicate upward CSF flow (bright signals in orange ROIs) at all levels (**a, b, d**) during IN. Spinal EV (**b,d**) and IJV (**c**) show simultaneous downward flow (dark signal in blue ROIs). Of note, flow in IVC (blue ROI, (**e**) subsides (grey signal). Downward CSF flow (dark signal) occurred at all levels (**a, b, d**) during EX. Flow in EV at C3 appears less dark (**b**), hence alleviated. Lumbar EVs (**d**) and IVC (**e**) show upward blood flow (bright). *Aqd =* aqueduct, *C3 *= cervical level 3, *L3* = lumbar level 3, *EV =* epidural veins, *IJV =* internal jugular vein, *IVC =* inferior vena cava.

Figure 1 displays original magnitude images and zoomed sections of corresponding velocity maps during forced inspiration and expiration. The examples cover the three slice positions at aqueduct (Aqd), spinal cervical level three (C3), and spinal lumbar level three (L3) of three representative subjects. Rows a, b and d refer to CSF flow measurements within the cranium and spinal canal, while rows c and e illustrate the regions of interest (ROI) for cervical and abdominal venous flow.

Magnitude images possess a high sensitivity to through-plane flow because the inflow of previously unsaturated spins into the measurement section increases its signal intensity relative to the saturated spins within the slice. On the other hand, dark and bright signals in velocity maps correspond to opposite flow directions with grey values representing zero movement, i.e. stationary tissue. In all sections, the occurrence of bright signals in velocity maps refers to upward flow, while dark signals represent downward flow.

**Figure 2:**
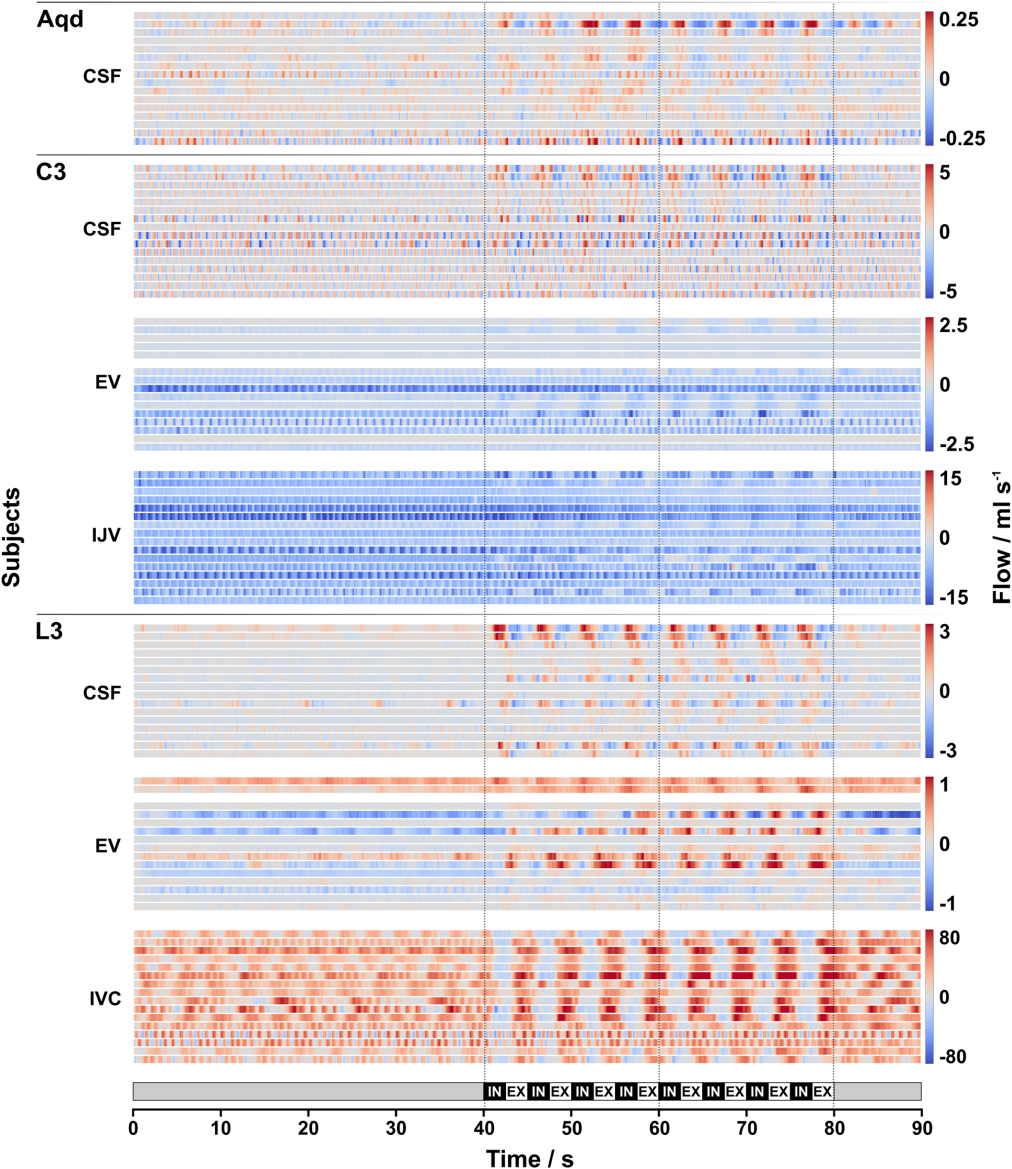
Individual CSF and venous flow. Color-coded flow rates (ml s^−1^) of all 16 subjects in 16 horizontal lines for each ROI during 90 s breathing protocol (bottom). Vertical lines mark start, middle and end of forced breathing. Note the different scaling for the various ROIs. **Normal breathing (0–40 s):** low CSF flow (blue = downward; red = upward) is regulated by cardiac pulsation (e.g., in Aqd and L3). Venous blood shows steady downward flow in EV and IJV with cardiac pulsatility. IVC flow is predominantly in upward direction with cardiac and respiratory influences (e.g., #1, #4, #5). Low flow at L3-EV is variable between subjects (e.g., opposite directionality between #10 and #5). **Forced breathing (40–80 s):** CSF and venous flow synchronized to respiration. During IN CSF moves predominantly upwards (various red shades), downward venous flow in C3-EV and IJV is modulated by IN (darker blue). Upward flow in IVC ceases or reverses (blue). During EX downward CSF movement (blue) prevails at all locations, venous flow decreases (lighter blue) at C3 and resumes upward directionality in IVC at L3 (red). Flow in L3-EV shows greater variability, however, respiratory modulations are observable (e.g. opposite behavior in subject #1 compared to #2). **Normal breathing (80–90 s):** rapid return of CSF and blood flow to the initial pattern. *Aqd* = aqueduct, *C3 =* cervical level 3, *L3 =* lumbar level 3, *EV =* epidural veins, *IJV =* internal jugular vein, *IVC =* inferior vena cava, *IN =* inspiration, *EX* = expiration.

**Figure 3:**
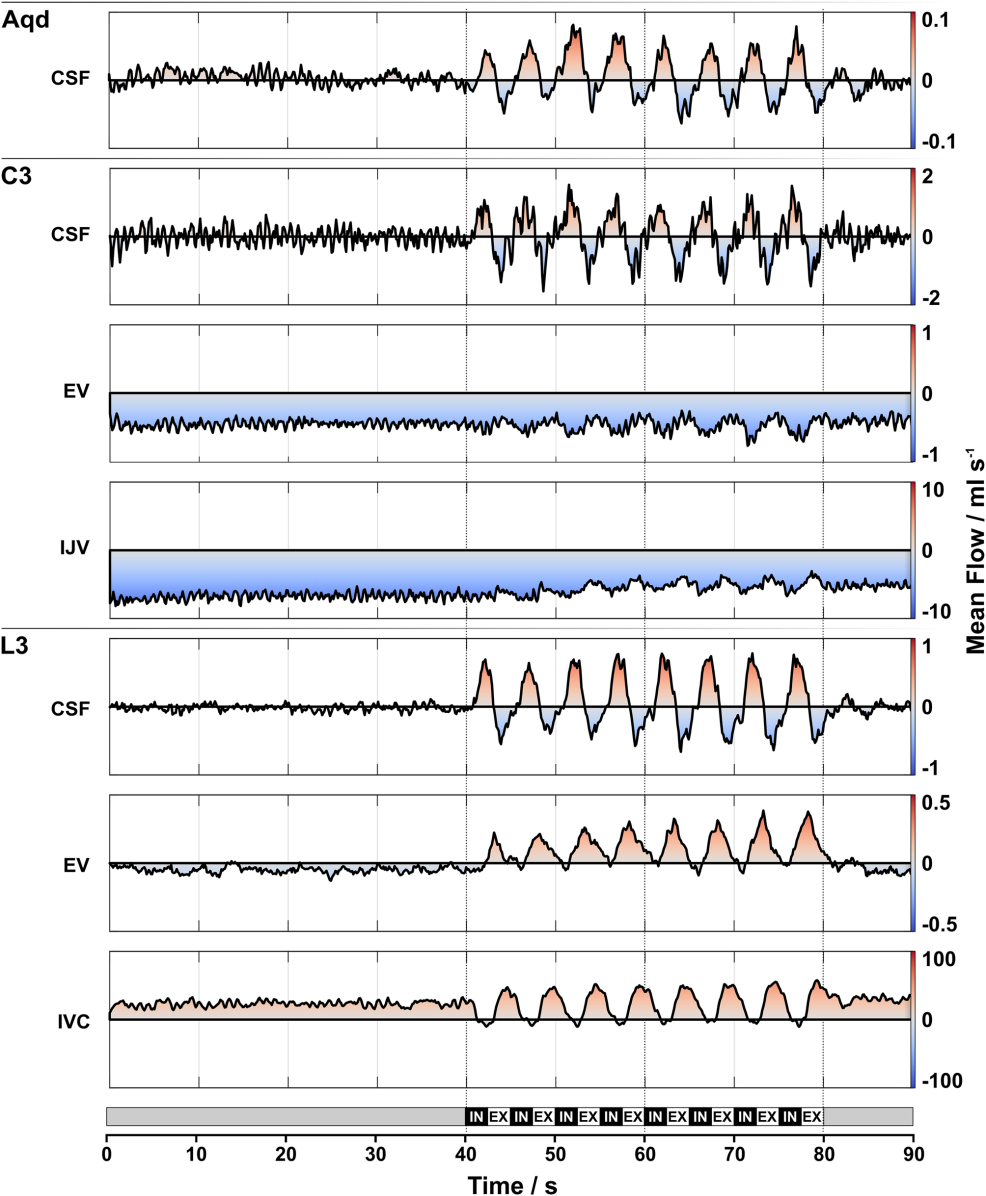
Mean CSF and venous flow. Color-coded mean flow rates (ml s^−1^) of CSF and venous blood averaged across subjects at all locations during the 90 s breathing protocol (bottom). The procedure largely removes the cardiac component. Note the different scaling for the various ROIs. **Normal breathing (0–40 s):** low CSF flow with cardiac pulsatility. Venous flow in C3-EV and IJV remains constantly negative (blue). IVC shows upward (red) flow. Mean L3-EV flow is close to zero with a tendency towards the downward direction (light blue). **Forced breathing (40–80 s):** synchronous flow of CSF and venous blood following respiratory modulations. CSF moves upwards with IN in all ROIs (red) and downwards during EX (blue). Venous blood in the vessels at C3 shows downward flow with only small modulations. In L3-IVC flow modulations are more pronounced i.e. assuming a decrease during IN and an increase during EX. Flow in L3-EV yields a similar behavior. **Normal breathing (80–90 s):** rapid return of CSF and blood flow to the initial pattern. *Aqd* = aqueduct, *C3 =* cervical level 3, *L3 =* lumbar level 3, *EV =* epidural veins, *IJV =* internal jugular vein, *IVC* = inferior vena cava, *IN =* inspiration, *EX =* expiration.

CSF and venous blood flow values over time at the Aqd as well as C3 and L3 for all 16 subjects are presented as a heat map in Fig. 2. Color-coded flow rates averaged across subjects are shown in Fig. 3 and flow rates averaged across time frames for the individual subjects are given in Suppl. Fig. 1. No flow signal could be detected in epidural veins at C3 (C3 EV) for subject #6 and in epidural veins at L3 (L3 EV) for subject #3.

As a general observation, CSF and venous blood flow reveal only minor respiration-driven dynamics during normal breathing. Forced respiration on the other hand, induced high-amplitude flow modulations at all locations in parallel to the breathing protocol (Figs. 2, 3). In the CSF system forced inspiration elicits an increase of flow in cranial direction (coded in red), i.e. at the Aqd, C3 and L3, whereas forced expiration causes a caudal CSF movement (coded in blue). The downward venous blood flow in the upper body (C3-EV, internal jugular veins at C3 (C3-IJV)) yields respiratory modulation with the onset of forced breathing. In the inferior vena cava (IVC) of the lower body the steady upward flow decreases during forced inspiration and rapidly increases again during forced expiration. Although flow in the EV at L3 shows great interindividual variability, respiratory modulations emerge during forced breathing parallel to those of the IVC.

**Figure 4:**
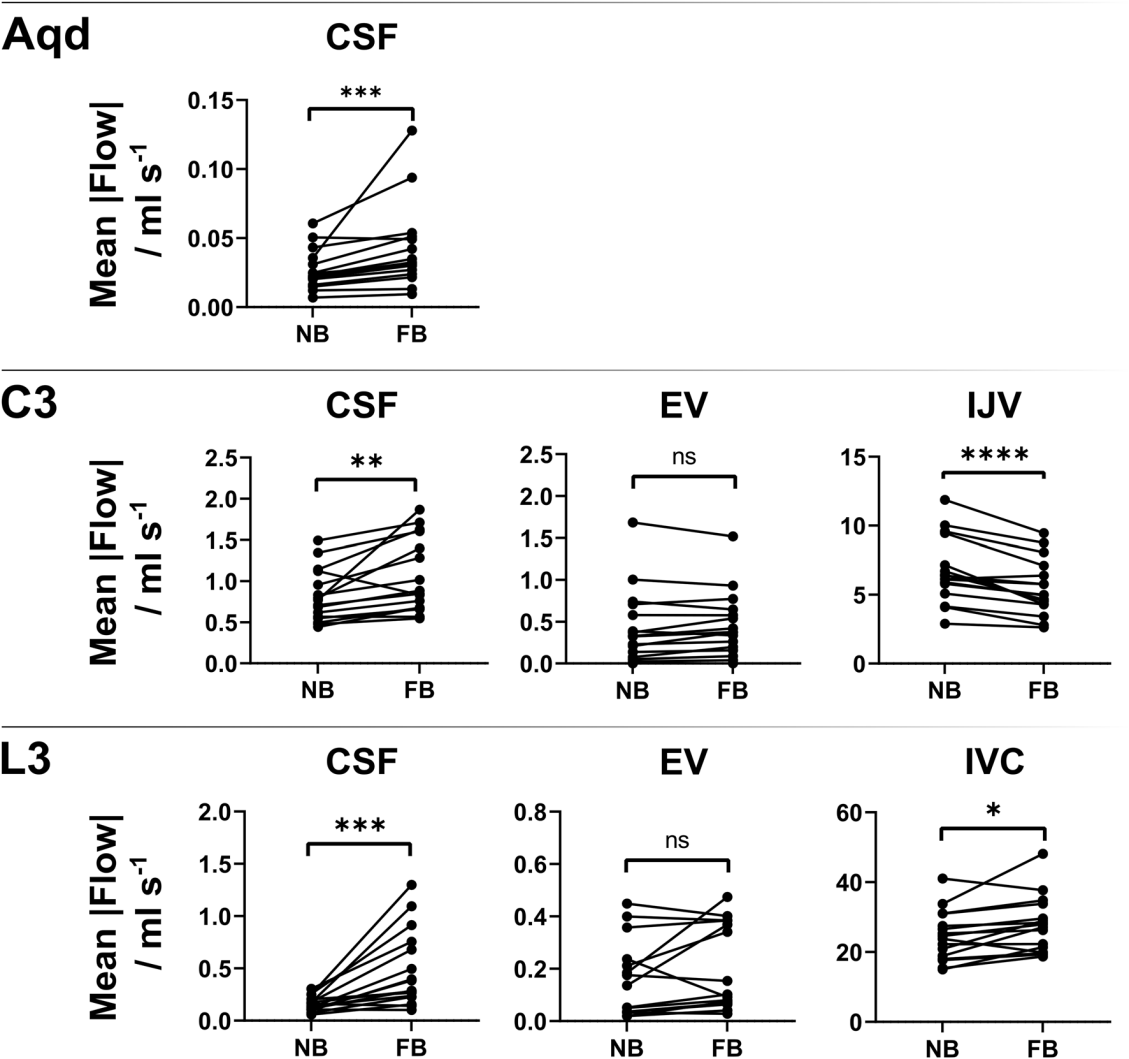
Difference of flow between normal and forced respiration. Paired t-test results at Aqd C3 (CSF, EV, IJV) and at L3 (CSF, EV, IVC). Each data point represents mean magnitude flow across all 320 time frames. Note the significant increase in CSF flow between normal and forced respiration at all location. Venous flow in lower body (L3-IVC) increases significantly with forced respiration while in upper body (C3-IJV) it decreases. In C3- and L3-EV no significant differences in flow can be detected. *Aqd =* aqueduct, *C3 =* cervical level 3, *L3 =* lumbar level 3, *EV =* epidural veins, *IJV* = internal jugular vein, *IVC =* inferior vena cava, *R/C* = respiration/cardiac, *NB =* normal breathing, *FB =* forced breathing, *ns =* not significant.

Figure 4 illustrates the temporal average of flow magnitude (mean across time frames) at all locations for each subject. The mean and SD across all subjects during normal and forced respiration and corresponding p-values of the paired t-tests are listed in Table 1. The average of the magnitude of CSF flow increases significantly during forced respiration at all locations (Aqd, C3, L3). Venous magnitude flow decreases significantly at C3-IJV and increases at L3-IVC with forced respiration, whereas no significant difference was found in EV (at C3, L3) compared to normal breathing.

**Table 1:** Average of flow magnitude during normal and forced breathing, mean ± SD across subjects.

ROI	Average of flow magnitude (ml s^−1^)		
	Normal	Forced	p-value
Aqd	0.02 ± 0.01	0.04 ± 0.03	0.001*
C3 CSF	0.83 ± 0.36	1.08 ± 0.51	0.016*
C3 EV	0.45 ± 0.49	0.49 ± 0.41	0.175
C3 IJV	6.8 ± 2.6	5.5 ± 2.2	0.0008*
L3 CSF	0.15 ± 0.07	0.50 ± 0.38	0.004*
L3 EV	0.19 ± 0.16	0.24 ± 0.17	0.175
L3 IVC	25.5 ± 7.5	28.7 ± 8.7	0.047*

Aqd = aqueduct; C3 = cervical level 3; L3 = lumbar level 3; EV = epidural veins; IJV = internal jugular vein; IVC = inferior vena cava; normal = normal breathing; forced = forced breathing; * = p < 0.05.

### Coupling of CSF and venous flow

**Figure 5:**
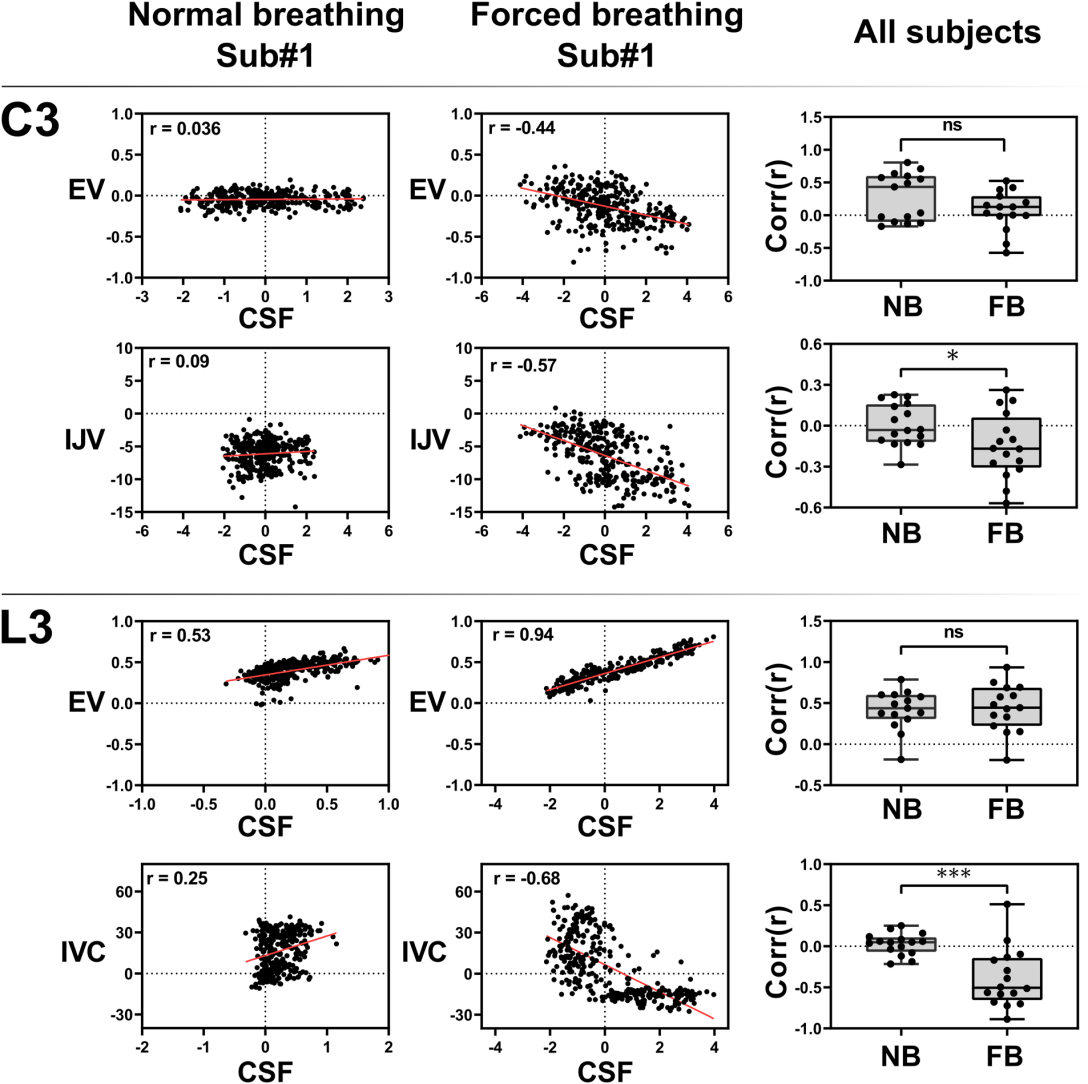
Correlation between CSF and venous flow. Scatter plots show relation between C3-CSF v/s EV, C3-CSF v/s IJV, L3-CSF v/s EV, L3-CSF v/s IVC during normal breathing (left column) and forced breathing (middle column) for subject #1. Red lines represent the linear regression and ‘r’ is the correlation coefficient. Right column displays the Pearson’s correlation coefficient of all subjects and p-values of student’s t-tests. Significance is reached for C3-CSF v/s IJV and L3-CSF v/s IVC but not for C3-CSF v/s EV and L3-CSF v/s EV. *C3* = cervical level 3, *L3 =* lumbar level 3, *EV =* epidural veins, *IJV =* internal jugular vein, *IVC =* inferior vena cava, *Corr(r)* = Pearson’s correlation coefficient, *NB* = normal breathing, *FB* = forced breathing, *ns* = not significant, *Sub* = subject.

The results of a correlation analysis between CSF and venous flow time series in both upper body and lower body are displayed in Fig. 5. Individual correlation coefficients for normal and forced breathing were calculated for C3-CSF v/s EV, C3-CSF v/s IJV, L3-CSF v/s EV, and L3-CSF v/s IVC. For all subjects and all locations (Fig. 5 right column) forced respiration tends to cause a stronger correlation between CSF and venous flow as compared to normal breathing. For most subjects flow during forced breathing at C3-CSF and L3-CSF is negatively correlated with C3-IJV and L3-IVC, respectively. In contrast, L3-CSF and L3-EV flow shows a positive correlation for most of the subjects (13/16). The paired t-tests reveal significant changes in correlation coefficients for normal and forced breathing, hinting at forced breathing to synchronize CSF and venous flow in the large vessels. The change for CSF v/s EV is not significant at both locations.

### Respiratory and cardiac contributions to flow spectrum (frequency analysis)

**Figure 6:**
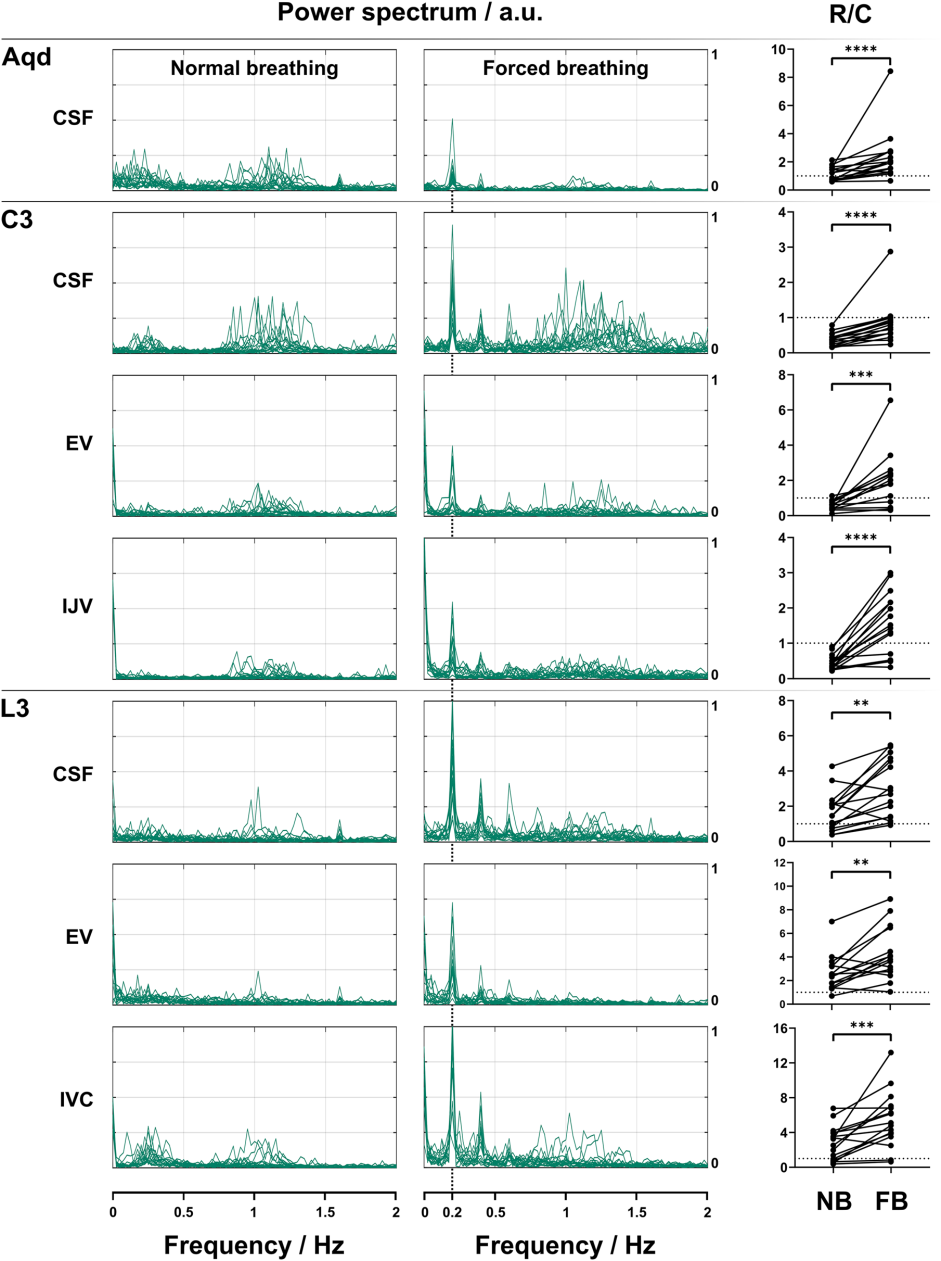
Frequency analysis of CSF and venous flow. Frequencies (Hz) spectrum of flow signals of 16 subjects during normal (left column), forced breathing (middle column) and power ratio R/C (right column). Note the predominance of frequencies corresponding to heart rates during normal breathing for CSF (Aqd, C3) and C3 venous flow and R/C values < 1 (right column). During forced breathing frequencies of 0.2 Hz (5 s = one breathing cycle in the protocol) and 0.4 Hz (2.5 s = half a breathing cycle) dominated at all locations in both fluid systems with corresponding R/C values > 1 except for C3-CSF. Here, the frequencies related to heart rates and the R/C remained < 1. In all localizations R/C values increased significantly from normal to forced breathing. *Aqd =* aqueduct, *C3 =* cervical level 3, *L3 =* lumbar level 3, *EV =* epidural veins, *IJV =* internal jugular vein, *IVC =* inferior vena cava, *R/C =* respiration/cardiac, *NB =* normal breathing, *FB =* forced breathing, *ns =* not significant.

Figure 6 depicts the frequency spectra of the flow dynamics of each subject in all measured regions of the venous and CSF system during normal (left column) and forced breathing (middle column). During normal breathing, the maximum frequency components for CSF and venous flow at C3 are predominantly located between 0.9 and 1.5 Hz and thus in the range of heart rates with corresponding R/C values < 1 (right column) which summarize these findings by the ratio of low (respiratory) and high (cardiac) frequency components. At L3, dominant frequencies of < 0.5 Hz correspond to respiratory modulations for CSF as well as for EV flow resulting in R/C values > 1. In the Aqueduct, no predominance of cardiac or respiratory modulations is found and hence the R/C during normal breathing is close to 1.

During forced breathing the prevailing frequency component shifted to 0.2 Hz which matches the periodicity of the breathing protocol. The corresponding R/C values were > 1 at all locations except for C3-CSF where it remained < 1. The increase of the R/C value from normal to forced breathing is significant in all positions (Fig. 6).

### ROI sizes during normal and forced breathing

The temporal evolution of the individual ROI sizes (mm^2^) for all 16 subjects is depicted in Suppl. Fig. 2. ROI areas in C3-IJV and L3-IVC slightly fluctuate in parallel to forced breathing. In 6 of the 7 ROIs, no consistent temporal alterations were observed during normal or forced respiration and the comparison between both parts of the protocol reveals no significant changes (Suppl. Table 1). Only the ROI of the IJV at C3 shows a significant decrease in size (p = 0.0012) during 40 s of forced breathing.

## Discussion

The central observation of this work is the fact that the dynamics of CSF and venous blood flow vary distinctly between normal and forced breathing and thus confirm the strong dependence of both systems on respiration. In contrast to normal breathing where cardiac driven frequency components prevail, the beginning of deep respiration causes immediate adherence of fluid dynamics to that driving force. As demonstrated by the shift of the main frequency component of CSF flow, the transition from normal to forced breathing promptly causes a switch from cardiac- to respiration-related dynamics in all positions except for C3-CSF (Fig. 6). Moreover, forced inspiration elicits a distinct upward surge of CSF from the lumbar region up to the intracranial aqueduct in line with previous findings. In contrast, forced expiration leads to reversed, downward flow. Venous outflow from the head/neck region is also modulated by forced respiration which so far has only been demonstrated for the cervical epidural veins^11,18^. Simultaneously, the steady venous flow from the lower body up towards the heart abates at the onset of forced inspiration and rises quickly afterwards throughout expiration.

CSF flow shows rapid respiration-dependent adjustments. Upward surge of CSF during forced inspiration is considered to balance the simultaneously enhanced venous pooling out of the head/neck region which is driven by lowering of the intrathoracic pressure in accordance with the Monro–Kellie doctrine^12^. Transmissions of intrathoracic and also intraabdominal pressure changes into the bony spinal canal are assumed to be facilitated by the presence of abundant connections from the extraspinal paravertebral venous plexus through the intervertebral foramina to the epidural spaces^19,20^. As a result, the high-capacity, valveless epidural venous plexus have been found to be kept open by negative pleural and epidural transmural pressures^14,19,21,22^. However invasive in vivo measurements of intrathoracic or intraabdominal pressures were not obtained in this study.

The amplified CSF movements during forced breathing resulted in a significant increase of average magnitude flow at all positions compared to normal breathing. As no significant variations in CSF ROI sizes occurred over time the rise of flow rates is ascribed to changes of flow velocities (Suppl. Table 1, Suppl. Fig. 2). Moreover, the shift of frequency components towards respiratory rates leads to a significant increase of R/C values thus corroborating deep breathing as the prevailing driving force.

In support of our findings, contributions of deep breathing to CSF velocities and flow rates have been shown in several previous real-time flow MRI studies albeit mainly at the aqueduct and foramen magnum. For the aqueduct Takizawa et al.^23^ reported a greater amount of CSF displacement by respiratory than cardiac contributions, however, at lower velocities. A recent computational study conducted by Vinje et al.^24^ demonstrated that small respiratory-induced pulsations of the ICP gradient induce CSF flow volumes dominating the cardiac component. Also Yildiz et al.^25^ reported in single slice real-time flow MRI measurements at the foramen magnum smaller R/C ratios during natural breathing compared to deep breathing.

Venous flow in the upper body was measured in the IJV and cervical EV as well as in the IVC and lumbar EV of the lower body’s venous system. The constant venous drainage from the head/neck region through IJV and EV alters distinctly with forced breathing and flow adheres to respiratory-driven patterns in agreement with previous studies^11,26^. The start of forced inspiration perturbs the steady venous return which prevails in the IVC during normal breathing. Instantaneously, flow ceases and even briefly reverses downward. During forced respiration the frequency of the main venous flow component adhered to the breathing protocol.

The average of venous flow magnitude showed a trend towards higher numbers in cervical and lumbar EV and IVC reaching significance only in the latter (Table 1). At the cervical level, we observed respiratory modulations in both IJV and EV as described by Epstein et al.^27^. While in the EV, flow only tended to increase over 40 s forced breathing, averaged flow magnitude in the IJV (Table 1) and their ROI areas decreased significantly (Suppl. Table 1). To our knowledge real-time cervical venous flow has not been studied during forced breathing in supine position so far. One of the reasons for the discrepancy between outflow through the IJV and EV could be hypocapnia as a probable hyperventilatory effect. As one limitation, we did not control CO_2_ partial pressures, but explained signs of hyperventilation to the subjects and instructed them specifically to avoid these. Furthermore, hypocapnia affects cardiac output, arterial flow and to lesser extent cerebral blood volume^28–30^. Hence, its influence on the venous system might only be secondary and would be expected to occur in all veins. Flow differences between these two cerebrovenous drainage pathways have so far been found in relation to postural changes. Multiple studies assigned the dominance of IJV drainage to the supine position and pointed to the EV systems as the major pathway in the upright position^31^. The bony spinal canal protects the thin valveless venous plexus from direct exposure to the thoracic and abdominal pressure changes and constant negative pleural and epidural pressures as well as bridging trabeculae within their lumen prevent the collapse of the vessels^14,19,20,31,32^. In contrast, forced breathing might expose the IJV to various pressure changes along its course in the neck and at the entrance to the thoracic cavity.

Venous flow in the lower body was explored in the IVC. The steep decrease and reversal of flow in that vessel during forced breathing resulted in significantly increased average flow magnitudes (Table 1) while ROI size remained vastly unchanged (Suppl. Table 1). A similar flow pattern has been reported by Joseph et al.^33^ applying real-time flow MRI. Older studies based on clinical observations and invasive in vivo measurements of intracaval pressure and blood velocity also reported a paradoxical reduction of IVC flow parallel to deep inspiration^34,35^. The simultaneous increase of abdominal pressure and reduction of intrathoracic pressure changed the IVC configuration from distended to collapsed at the level of the diaphragm as demonstrated by X-ray. The authors correlated the findings to a sharp anatomical constriction of the large vein where it passes through the diaphragm in postmortem casts and to the principles of flow through collapsible tubes^26,34^. Small sizes and irregular anatomy of the epidural venous plexus usually impede in vivo flow MRI studies of lumbar EV. Moreover, the variability of flow in L3-EV is particularly high during normal breathing, e.g. see Fig. 2. Individual venous plexus anatomy as well as factors such as breathing performance might play important roles. The results of 15/16 healthy subjects reported here represent one of the largest cohorts for flow measurements under physiological conditions. A trend towards higher average flow magnitudes was seen during forced breathing not reaching significance (Table 1). Their dynamics parallel the behavior in the IVC with upward movements during expiration.

In recent years, clinically relevant disorders of CSF circulation and venous systems pathologies have increasingly been linked. The exact mechanisms which couple cerebral blood flow and CSF movement are up to this point not well understood. In our study, flow in the large veins yields a significantly negative correlation with corresponding CSF dynamics during forced breathing which is presumed to prompt sufficient intrathoracic and abdominal pressure changes (Fig. 5). Correlation coefficients showed a negative trend in C3-CSF v/s EV and a positive in L3-CSF v/s EV without reaching significance. This might be caused by a delayed response of flow in the EV to forced breathing or by a higher uncertainty of flow measurements due to the delicate vessel sizes and thus smaller amount of flow. Furthermore, pressure conditions in the epidural space have been demonstrated to vary along the spine which could result in less uniform flow behavior^35^.

Taken together, this study provides in vivo evidence that deep breathing represents a physiological coupling of cranio-spinal fluid flow. Although the clinical impact warrants further investigations, an increasing number of clinical observations point to an important pathophysiological role. For example, compromised venous flow is considered one cause of IIH, where increased intracranial pressure leads to headaches and papilledema^36,37^. Intracranial obstruction of venous outflow i.e. due to transverse sinus stenosis or extracranial IJV obstruction have been accounted for perturbed intracranial pressure regulation and CSF circulation^36,38^. Moreover, cerebral venous hypertension as a consequence of sleep-disordered breathing is discussed as one of the key factors for the development of normal pressure hydrocephalus, leading to ventriculomegaly and dementia^39^. In infants, Shulman et al.^15^ related elevated pressure in the sagittal sinus to the occurrence of hydrocephalus. Thus, disturbances of the venous-CSF coupling mechanism might indeed represent a key in the pathophysiology of related CSF disorders.

## Conclusions

In contrast to normal breathing, forced respiration leads to pronounced increases and a prompt synchronization of flow dynamics in CSF and venous systems. The lumbar caval and epidural venous upward surge during free breathing abates with the onset of forced inspiration and rises during ensuing exhalation—a pattern opposite to that of venous flow in the upper body part. Spinal and intracranial CSF, on the other hand, move uniformly upwards during forced inhalation as previously described. Our results provide evidence that deep respiration couples interdependent venous and brain fluid flow—most likely mediated by intrathoracic and intraabdominal pressure changes. Further insights into the tight interplay between CSF and venous fluid dynamics will expand our understanding of the pathophysiology of human diseases with CSF flow disturbances such as hydrocephalus and facilitate the development of more specific therapeutic options.

## Methods

### Subjects

Sixteen healthy volunteers (6 females, 10 males, age, 28 ± 5; (mean ± SD); height 178 ± 8 cm, weight 73 ± 12 kg, BMI 23 ± 2.4 kg/m^2^) without contraindication for MRI and no known illness were enrolled. The study was approved by the institutional review board of the University Medical Center Göttingen (#18/2/14) and written informed consent was obtained from each subject prior to MRI. The study was in compliance with the Declaration of Helsinki.

### Study design

Real-time flow MRI was performed in three transversal cross-sections covering intracranial and spinal CSF and the venous systems at the Aqd, C3 and L3. ROIs for the analysis of CSF dynamics were placed in the Aqd  and spinal subarachnoid spaces at C3 and L3 as outlined in Fig. 1a–e. For corresponding venous flow determinations ROIs were drawn around the IJVs (Fig. 1c) with the stronger flow signal in the neck region and around the lumbar IVC (Fig. 1e). The venous plexus expanding in the epidural spaces inside the entire vertebral column (internal vertebral venous plexus) commonly forms prominent orthogonal veins at C3 which resemble a rope ladder. The more prominent EV was selected for the ROI analysis (Fig. 1b). The lumbar venous plexus forms a spacious mesh rendering epidural vessels less well identifiable. ROIs in that region were defined around flow signals detectable in the epidural space ventral to the CSF space (Fig. 1d).

All subjects were examined in supine position and required to follow a visually presented breathing protocol. Timing and commands for deep inspiration and expiration were explained and trained beforehand as inspiration and exhalation had to occur gradually over 2.5 s periods. Furthermore, subjects were instructed to avoid hyperventilation. The breathing protocol started with 40 s of normal free breathing followed by 8 cycles of 2.5 s forced inspiration and 2.5 s forced expiration. The protocol concluded with another 10 s period of normal breathing summing up to a total of 90 s per scan (see Fig. 3, bottom line). Subjects were without any cardiac conditions, had pulse rates within normal age limits, while respiration was monitored via a belt fixed at the level of the diaphragm. Individual breathing performance and adherence to the protocol were evaluated by visual observation and measurements were repeated if deemed necessary. The flow data were acquired in the same order for all subjects and an individual study lasted about 40–50 min.

### Real-time phase-contrast flow MRI

All data were acquired on a 3 Tesla scanner (Magnetom Prisma Fit, Siemens Healthcare) using real-time phase-contrast flow MRI based on highly undersampled radial FLASH sequences^40,41^ with timing-optimized gradient design^42^. Quantitative velocity maps were obtained by a model-based reconstruction technique offering access to high spatiotemporal resolutions^43^. The field of view was 192 mm (Aqd, C3) or 256 mm (L3) and image matrix sizes were 256 or 320, resulting in in-plane resolutions of 0.75 × 0.75 and 0.8 × 0.8 mm^2^, respectively. Further MRI parameters were set as follows: slice thickness 5 mm, flip angle 10°, repetition time (TR) 5.68 ms, and echo time (TE) 4.61 ms (Aqd, C3) or 4.57 ms (L3). In either case, two flow-encoded datasets were acquired with 11 radial spokes to keep a fixed temporal resolution of 125 ms per velocity map corresponding to a rate of 8 frames per second. The measurement protocol resulted in a total of 720 magnitude images and velocity maps, 320 images each during 40 s of normal and forced respiration followed by another 80 images during the final 10 s of normal breathing. The velocity encoding strength (VENC) was adapted according to the peak velocities of CSF or blood flow. While all CSF measurements as well as studies of EV at C3 and L3 exploited low VENC values of 10 to 30 cm s^−1^ (only for phase wraps in the EV), measurements were repeated with a higher VENC of 60 to 100 cm s^−1^ focussing on the large veins at C3 and L3. Measurements in the aqueduct and at C3 were conducted with a 64-channel head coil, while measurements at L3 used suitable elements of an 18-channel thorax coil and a 32-channel spine coil.

### Data analysis

Real-time flow MRI datasets were quantitatively analysed using CaFuR software (Fraunhofer Mevis, Bremen, Germany)^44^ designed to accomplish automatic segmentation of flow signals in real-time image series after manual definition of a single initial ROI, which then serves as a seeding image for dynamic segmentation. The determination of through-plane flow was based on both signal intensities in magnitude images and corresponding phase difference values in velocity maps (see Fig. 1, right columns for representative examples). Further data processing was performed using Matlab (Mathworks, Massachusetts, USA).

For a comparison of the two parts of the breathing protocol, flow rates and ROI sizes were presented as mean values averaged across time frames and grouped into normal (40 s) and forced (40 s) breathing. Prior to temporal averaging, the magnitude of each flow time series was taken to remove the flow’s directionality. Further statistical analysis then was performed on the averaged flow magnitude. After a Shapiro Wilk test to test for normal distribution across subjects, a paired t-test was employed to find differences in averaged flow magnitude rates and ROI sizes between normal and forced respiration. Alternatively, a Wilcoxon Signed Rank test was applied for not normally distributed data. Statistical significance was accepted at p values < 0.05.

Temporal synchronisation of CSF and venous flow was studied using Pearson’s correlations for each subject individually, to investigate whether CSF and venous system are coupled. Correlation coefficient pairs (from normal and forced breathing) of each subject were then used for a paired t-test to investigate the effect of respiration on synchronisation of CSF and venous flow.

To illustrate flow components related to respiration and cardiac pulsation, the time series data were analysed in the frequency domain. A power ratio analysis as described by Yildiz et al. determined the relative contribution of respiration versus cardiac (R/C) components of the flow data^25^. Here, R is defined as the area under curve of low frequencies (0–0.5 Hz) and C bases on a 0.5 Hz interval around the individual maximum frequency close to 1 Hz. R/C values larger than one thus indicate flow dynamics predominantly modulated by respiration and R/C values smaller than one a predominance of cardiac modulations. A paired t-test was applied to evaluate differences between forced and normal breathing.

### Ethics approval and consent to participate

The study was approved by the institutional review board of the University Medicine Göttingen ((#18/2/14) and written informed consent was obtained from each subject prior to MRI. The study was in compliance with the Declaration of Helsinki.

## Supplementary Information

Supplementary Figure 1.

Supplementary Figure 2.

Supplementary Table 1.

## Data Availability

The datasets used and/or analyzed during the current study are available from the corresponding author on reasonable request.

## Abbreviations

Aqd: Aqueduct
CSF: Cerebrospinal fluid
C3: Cervical spine at level 3
EX: Expiration
EV: Epidural veins
ICP: Intracranial pressure
IIH: Ideopathic intracranial hypertension
IJV: Internal jugular vein
IN: Inspiration
IVC: Inferior vena cava
L3: Lumbar spine at level 3
MRI: Magnetic resonance imaging
R/C: Ratio of respiratory and cardiac frequency components
SD: Standard deviation

## References

[1] Elvsashagen T (2019). Cerebral blood flow changes after a day of wake, sleep, and sleep deprivation. Neuroimage.

[2] Oreskovic D, Rados M, Klarica M (2017). Role of choroid plexus in cerebrospinal fluid hydrodynamics. Neuroscience.

[3] Xie L (2013). Sleep drives metabolite clearance from the adult brain. Science.

[4] Jiang Q (2017). Impairment of the glymphatic system after diabetes. J. Cereb. Blood Flow Metab..

[5] Wardlaw JM (2020). Perivascular spaces in the brain: Anatomy, physiology and pathology. Nat. Rev. Neurol..

[6] Bock HC, Dreha-Kulaczewski SF, Alaid A, Gartner J, Ludwig HC (2019). Upward movement of cerebrospinal fluid in obstructive hydrocephalus-revision of an old concept. Childs Nerv. Syst..

[7] Frahm J, Voit D, Uecker M (2019). Real-time magnetic resonance imaging: Radial gradient-echo sequences with nonlinear inverse reconstruction. Investig. Radiol..

[8] Greitz D, Franck A, Nordell B (1993). On the pulsatile nature of intracranial and spinal CSF-circulation demonstrated by MR imaging. Acta Radiol..

[9] Dreha-Kulaczewski S (2015). Inspiration is the major regulator of human CSF flow. J. Neurosci..

[10] Aktas G (2019). Spinal CSF flow in response to forced thoracic and abdominal respiration. Fluids Barriers CNS.

[11] Dreha-Kulaczewski S (2017). Identification of the upward movement of human CSF in vivo and its relation to the brain venous system. J. Neurosci..

[12] Greitz D (1992). Pulsatile brain movement and associated hydrodynamics studied by magnetic resonance phase imaging. The Monro-Kellie doctrine revisited. Neuroradiology.

[13] Nakada T, Kwee IL (2019). Fluid dynamics inside the brain barrier: Current concept of interstitial flow, glymphatic flow, and cerebrospinal fluid circulation in the brain. Neuroscientist.

[14] Ludwig HC (2021). Hydrocephalus revisited: New insights into dynamics of neurofluids on macro- and microscales. Neuropediatrics.

[15] Shulman K, Ransohoff J (1965). Sagittal sinus venous pressure in hydrocephalus. J. Neurosurg..

[16] Haacke EM, Beggs CB, Habib C (2012). The role of venous abnormalities in neurological disease. Rev. Recent Clin. Trials.

[17] Morris PP, Black DF, Port J, Campeau N (2017). Transverse sinus stenosis is the most sensitive MR imaging correlate of idiopathic intracranial hypertension. Am. J. Neuroradiol..

[18] Dreha-Kulaczewski S (2018). Respiration and the watershed of spinal CSF flow in humans. Sci. Rep..

[19] Henriques CQ (1962). The veins of the vertebral column and their role in the spread of cancer. Ann. R. Coll. Surg. Engl..

[20] Stringer MD, Restieaux M, Fisher AL, Crosado B (2012). The vertebral venous plexuses: The internal veins are muscular and external veins have valves. Clin. Anat..

[21] Nystrom EU, Blomberg SG, Buffington CW (1998). Transmural pressure of epidural veins in the thoracic and lumbar spine of pigs. Anesthesiology.

[22] Ludwig HC, Dreha-Kulaczewski S, Bock HC (2021). Neurofluids-deep inspiration, cilia and preloading of the astrocytic network. J. Neurosci. Res..

[23] Takizawa K, Matsumae M, Sunohara S, Yatsushiro S, Kuroda K (2017). Characterization of cardiac- and respiratory-driven cerebrospinal fluid motion based on asynchronous phase-contrast magnetic resonance imaging in volunteers. Fluids Barriers CNS.

[24] Vinje V (2019). Respiratory influence on cerebrospinal fluid flow—A computational study based on long-term intracranial pressure measurements. Sci. Rep..

[25] Yildiz S (2017). Quantifying the influence of respiration and cardiac pulsations on cerebrospinal fluid dynamics using real-time phase-contrast MRI. J. Magn. Reson. Imaging.

[26] Wexler L, Bergel DH, Gabe IT, Makin GS, Mills CJ (1968). Velocity of blood flow in normal human venae cavae. Circ. Res..

[27] Epstein HM, Linde HW, Crampton AR, Ciric IS, Eckenhoff JE (1970). The vertebral venous plexus as a major cerebral venous outflow tract. Anesthesiology.

[28] Laffey JG, Kavanagh BP (2002). Hypocapnia. N. Engl. J. Med..

[29] Zhang Z, Guo Q, Wang E (2019). Hyperventilation in neurological patients: From physiology to outcome evidence. Curr. Opin. Anaesthesiol..

[30] Tercero J (2021). Effects on cerebral blood flow of position changes, hyperoxia, CO2 partial pressure variations and the Valsalva manoeuvre: A study in healthy volunteers. Eur. J. Anaesthesiol..

[31] Valdueza JM, von Munster T, Hoffman O, Schreiber S, Einhaupl KM (2000). Postural dependency of the cerebral venous outflow. Lancet.

[32] Groen RJ (2005). Morphology of the human internal vertebral venous plexus: A cadaver study after latex injection in the 21–25-week fetus. Clin. Anat..

[33] Joseph AA, Voit D, Frahm J (2020). Inferior vena cava revisited—Real-time flow MRI of respiratory maneuvers. NMR Biomed..

[34] Gardner AM, Turner MJ, Wilmshurst CC, Griffiths DJ (1977). Hydrodynamics of blood flow through the inferior vena cava. Med. Biol. Eng. Comput..

[35] Usubiaga JE, Moya F, Usubiaga LE (1967). Effect of thoracic and abdominal pressure changes on the epidural space pressure. Br. J. Anaesth..

[36] Ball AK, Clarke CE (2006). Idiopathic intracranial hypertension. Lancet Neurol..

[37] Mollan SP (2018). Idiopathic intracranial hypertension: Consensus guidelines on management. J. Neurol. Neurosurg. Psychiatry.

[38] Wilson MH (2016). Monro-Kellie 2.0: The dynamic vascular and venous pathophysiological components of intracranial pressure. J. Cereb. Blood Flow Metab..

[39] Roman GC, Jackson RE, Fung SH, Zhang YJ, Verma AK (2019). Sleep-disordered breathing and idiopathic normal-pressure hydrocephalus: Recent pathophysiological advances. Curr. Neurol. Neurosci. Rep..

[40] Joseph AA (2012). Real-time phase-contrast MRI of cardiovascular blood flow using undersampled radial fast low-angle shot and nonlinear inverse reconstruction. NMR Biomed..

[41] Unterberger MJ, Holzapfel GA (2014). Advances in the mechanical modeling of filamentous actin and its cross-linked networks on multiple scales. Biomech. Model Mechanobiol..

[42] Bernstein MA, Shimakawa A, Pelc NJ (1992). Minimizing TE in moment-nulled or flow-encoded two- and three-dimensional gradient-echo imaging. J. Magn. Reson. Imaging.

[43] Tan Z (2017). Model-based reconstruction for real-time phase-contrast flow MRI: Improved spatiotemporal accuracy. Magn. Reson. Med..

[44] Chitiboi, T. *et al*. Context-based segmemtation and analysis of multi-cycle real-time cardiac MRI. In *IEEE International Symposium on Biomedical Imaging*, 943–946 (2014).

