# Supplementary material for: Deep breathing couples CSF and venous flow dynamics
Source: Sci Rep. 2022 Feb 16;12:2568. doi: 10.1038/s41598-022-06361-x (PMC8850447; doi:10.1038/s41598-022-06361-x)
Supplement: Supplementary file 1 — Supplementary Figure 1. [file 41598_2022_6361_MOESM1_ESM.docx]

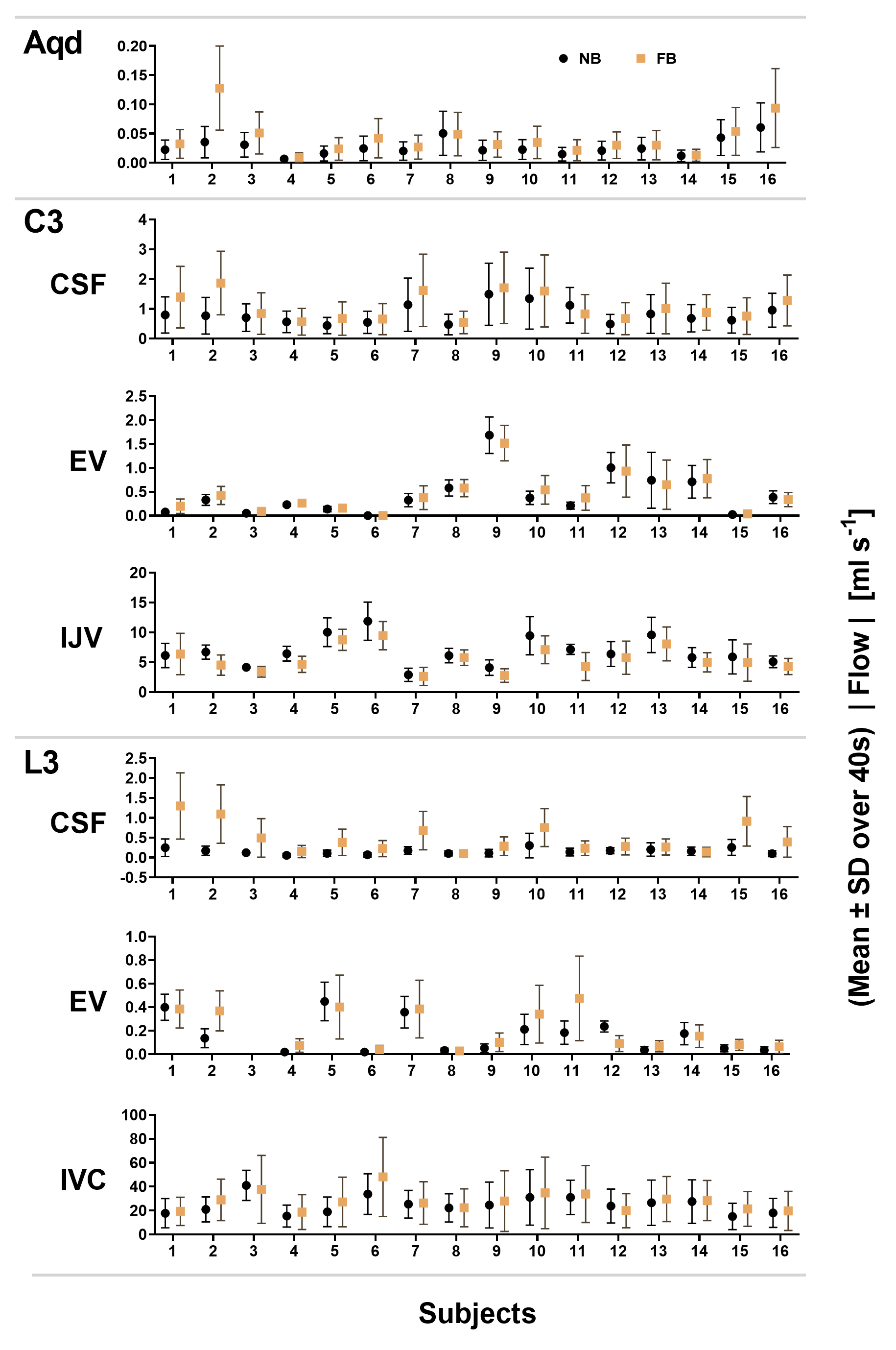


**Supplemental Figure 1. Absolute values of flow rates averaged across time frames for all subjects.**

Absolute values of flow rates (ml s^-1^) are averaged across the time frames for normal (black) and forced breathing (orange) for all 16 subjects individually. Student’s t-tests were applied to check for significant differences (p>0.05). Not significant differences between flow rates are marked. Aqd = aqueduct; C3 = cervical level 3; L3 = lumbar level 3; EV = epidural veins; IJV = internal jugular vein; IVC = inferior vena cava; NB = normal breathing; FB = forced breathing; ns = not significant.
