# Supplementary material for: Deep breathing couples CSF and venous flow dynamics
Source: Sci Rep. 2022 Feb 16;12:2568. doi: 10.1038/s41598-022-06361-x (PMC8850447; doi:10.1038/s41598-022-06361-x)
Supplement: Supplementary file 2 — Supplementary Figure 2. [file 41598_2022_6361_MOESM2_ESM.docx]

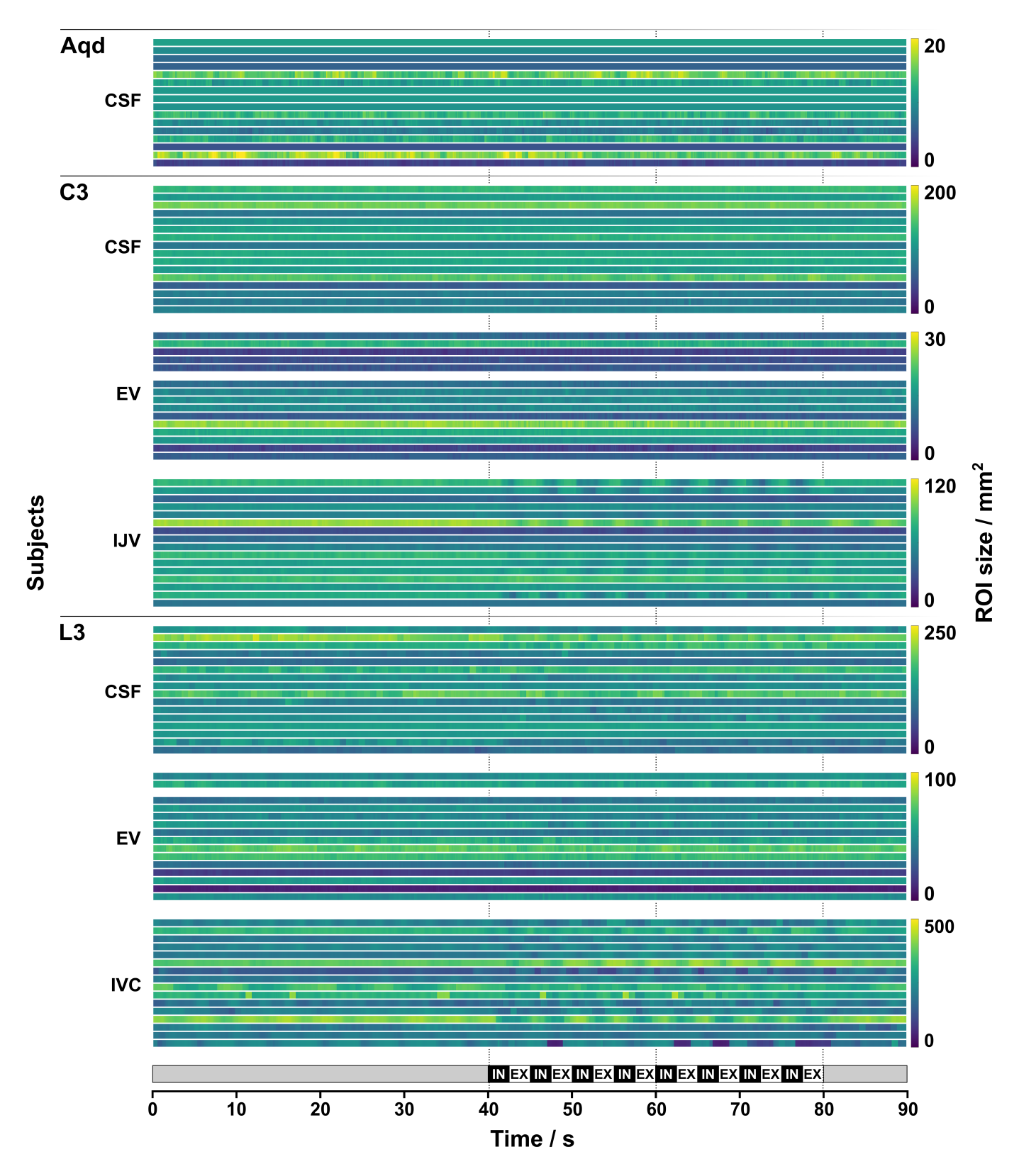


**Supplemental Figure 2. ROI sizes during normal and forced breathing.**

Color-coded sizes of all ROIs (mm^-2^) of the 16 subjects in 16 horizontal lines during the 90 s breathing protocol (bottom). Vertical lines mark start, middle and end of forced breathing. Note the different scaling of the ROIs. In some subjects cardiac-related variations are discernable. ROI sizes in C3 IJV and L3 IVC fluctuate to variable extent parallel to forced breathing. A trend towards smaller sizes can be observed in C3 IJV towards the end of the protocol. Aqd = aqueduct; C3 = cervical level 3; L3 = lumbar level 3; EV = epidural veins; IJV = internal jugular vein; IVC = inferior vena cava; IN = inspiration, EX = expiration.
