# Supplementary material for: Deep breathing couples CSF and venous flow dynamics
Source: Sci Rep. 2022 Feb 16;12:2568. doi: 10.1038/s41598-022-06361-x (PMC8850447; doi:10.1038/s41598-022-06361-x)
Supplement: Supplementary file 3 — Supplementary Table 1. [file 41598_2022_6361_MOESM3_ESM.docx]

**Supplemental Table 1: ROI sizes during normal and forced breathing.**

ROI sizes (mm^-2^, mean ± SD) averaged across 40 s (0-40 s) of normal and 40 s (40-80 s) of forced breathing, respectively. Aqd = aqueduct; C3 = cervical level 3; L3 = lumbar level 3; EV = epidural veins; IJV = internal jugular vein; IVC = inferior vena cava; normal = normal breathing; forced = forced breathing; * = p < 0.05.

| ROI | ROI size (mm^-2^) | | |
| --- | --- | --- | --- |
|  | normal | forced | p-value |
| Aqd | 10.0 ± 3.5 | 10.0 ± 3.3 | 0.598 |
| C3 CSF | 106 ± 27 | 107 ± 27 | 0.589 |
| C3 EV | 12.3 ± 5.5 | 12.2 ± 5.2 | 0.136 |
| C3 IJV | 62 ± 19 | 57 ± 16 | 0.0012* |
| L3 CSF | 131 ± 32 | 126 ± 32 | 0.139 |
| L3 EV | 47 ± 18 | 46 ± 17 | 0.773 |
| L3 IVC | 249 ± 77 | 243 ± 67 | 0.679 |
